# Piriform Fossa Injury during Calibration Tube Insertion in Laparoscopic Sleeve Gastrectomy

**DOI:** 10.3390/jcm12113824

**Published:** 2023-06-02

**Authors:** Taiki Nabekura, Takashi Oshiro, Kotaro Wakamatsu, Natsumi Kitahara, Yuki Moriyama, Kengo Kadoya, Ayami Sato, Tomoaki Kitahara, Tasuku Urita, Yu Sato, Masaru Tsuchiya, Shinich Okazumi

**Affiliations:** Department of Surgery, Toho University Sakura Medical Center, Sakura 285-8741, Japan; taiki.nabekura@med.toho-u.ac.jp (T.N.);

**Keywords:** laparoscopic sleeve gastrectomy, bariatric and metabolic surgery, complication, iatrogenic piriform fossa and esophageal injury, orogastric calibration tube

## Abstract

Piriform fossa and/or esophageal injuries caused by calibration tubes are relatively rare and remain unelucidated. Herein, we report the case of a 36-year-old woman with morbid obesity, sleep apnea, and menstrual abnormalities who was scheduled to undergo laparoscopic sleeve gastrectomy (LSG). We inserted a 36-Fr Nelaton catheter made of natural rubber as a calibration tube during the surgery. However, excessive resistance was observed. We confirmed a submucosal layer detachment approximately 5 cm from the left piriform fossa to the esophagus using intraoperative endoscopy. Additionally, LSG was performed using an endoscope as the guiding calibration tube. We inserted a nasogastric tube under endoscopy with a guidewire before completing the surgery, hoping for a guiding effect on the saliva flow. After 17 months, the patient had successfully lost weight postoperatively without complaints of neck pain or discomfort during swallowing. Therefore, in cases where the damage is limited to the submucosal layer, as in this case, conservative therapy should be considered; this is similar to the concept of endoscopic submucosal dissection not requiring suture closure. This case highlights the risk of iatrogenic injuries to the piriform fossa and/or esophagus during LSG and the importance of careful calibration tube insertion to prevent them.

## 1. Introduction

Bariatric metabolic surgery (BMS) is the most effective treatment for severe obesity and other obesity-related comorbidities. Laparoscopic sleeve gastrectomy (LSG), which is characterized by a simpler surgical technique than other BMSs, such as Roux-en-Y gastric bypass and sleeve gastrectomy with duodenojejunal bypass, is the most popular BMS according to the 7th IFSO Global Registry Report [1]. In Japan, the number of BMSs being performed has increased in recent years, mostly due to LSG [2], and a report on its efficacy and safety in the Japanese population has been published [3].

In LSG, a calibration tube with a diameter of >36 Fr is inserted orally to ensure the proper size of the sleeve-shaped stomach [4]. Due to the characteristic post-resection shape of the remnant stomach, patients experience unique complications post-LSG that differ from other BMSs, such as gastric torsion and flexion, uncontrollable gastroesophageal reflux disease (GERD), and leakage mainly in the region near the esophagogastric junction [5]. These complications are widely reported, and most surgeons who perform BMS recognize these issues [6,7,8]. However, the management of intraoperative iatrogenic piriform fossa and/or esophageal injuries caused by orogastric calibration tube insertion remains unclear. Piriform fossa and/or esophageal iatrogenic injury during BMS is relatively rare but can be life-threatening with one previous report of death due to mediastinitis [9]. Therefore, the risk of injury should not be underestimated.

Herein, we report the case of an iatrogenic injury of the piriform fossa caused by calibration tube insertion and the clinical management of this rare complication of LSG.

## 2. Case Presentation

A 36-year-old woman with clinically severe obesity (height, 151.6 cm; weight, 97.3 kg; body mass index (BMI), 42.3 kg/m^2^ at the time of her first visit) suffering from obstructive sleep apnea syndrome (apnea–hypopnea index, 21.0/h) and menstrual abnormalities was referred to our hospital for LSG. We adopted a multidisciplinary approach, including pharmacotherapy for obesity-related comorbidities, physical activity, nutritional advice, and consultations with psychiatrists and psychologists, to rule out and support psychosocial problems and mental disorders, and determine eligibility for BMS. The patient underwent preoperative investigations to verify eligibility for general anesthesia and surgery, which included routine blood examinations, upper gastrointestinal endoscopy, computed tomography (CT), electrocardiogram, and respiratory function tests. No investigations revealed any significant findings. The upper gastrointestinal endoscopy procedure was conducted smoothly, with no anatomical abnormalities noted, and no substantial gastrointestinal lumen-compressing lesions were observed on CT imaging.

Based on these results and discussions with our multidisciplinary team, we decided to perform LSG.

The primary surgeon had 8 years of experience as a surgeon and had previously performed as the primary surgeon in 24 BMSs. All surgeons who participated in this case were established members of the BMS team, and standardized hospital procedures were followed. An experienced supervising physician was involved in this surgery as an assistant.

**Figure 1:**
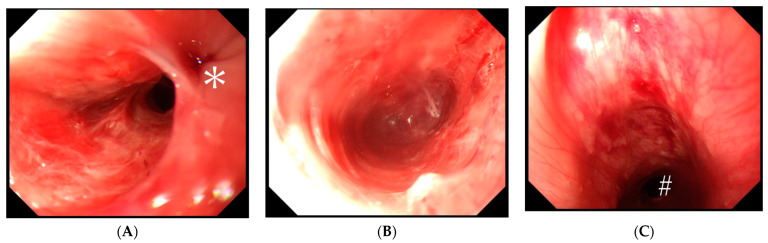
Intraoperative endoscopy images. Submucosal layer laceration from the left piriform fossa to the upper esophagus. The asterisk (*) indicates the esophageal orifice and the octothorpe (#) indicates the esophageal lumen. (**A**) The inlet of the submucosal layer laceration at the piriform fossa. (**B**) Laceration space with no active bleeding. (**C**) View from the upper esophagus. The laceration space is completely collapsed by endoscopic insufflation.

While administering general anesthesia, the patient experienced difficulty with intubation using a regular direct laryngoscope, which led to the need for a second attempt using a video-blade laryngoscope. Then, an experienced anesthesiologist blindly inserted a 36-Fr calibration tube, which is routinely used since there is no dedicated calibration tube for LSG in Japan, via the oral route. After the tip of the calibration tube was inserted approximately 10–12 cm, unusual backward resistance was felt, and hence, the tube was not inserted further. The calibration tube was immediately removed, and intraoperative upper gastrointestinal endoscopy using carbon dioxide was performed. Endoscopy revealed a submucosal layer laceration approximately 5 cm deep from the left piriform fossa to the upper esophagus, which was considered an iatrogenic injury caused by the calibration tube (Figure 1).

We consulted an otolaryngologist, who determined that the injury had likely not reached the mediastinum and it would be difficult to close the orifice. As the injury was limited to the submucosal layer and bleeding was not observed, we decided to continue with conservative follow-up to monitor the closure of the laceration. Circulation and respiratory status were stable; thus, we decided to continue the surgery. Sleeve gastrectomy was performed using an endoscope as a calibration tube with 6 cartridges of a 60 mm stapler. A full-layer continuous staple-line suture was performed using a nonabsorbable barbed suture. Before extubating, a 14-Fr nasogastric tube was placed using a guidewire during endoscopy. The operation time was 231 min, including the time required for the preparation and use of intraoperative endoscopy and consultation with the otolaryngology department, and the estimated blood loss was 5 mL.

**Figure 2:**
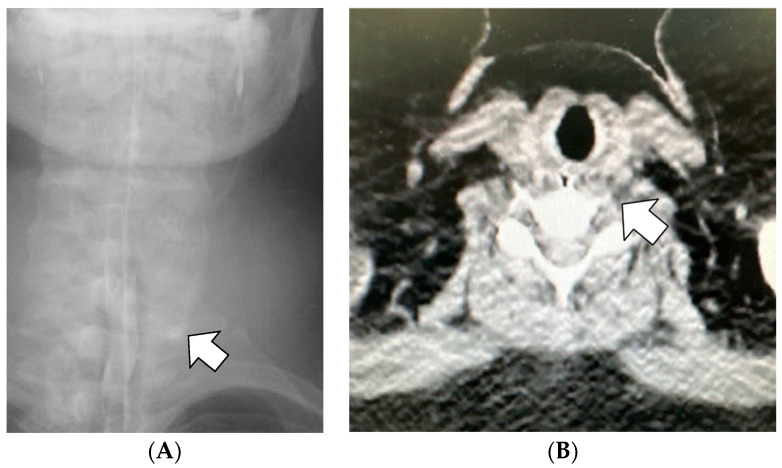
Fluoroscopy and contrast computed tomography (CT) images on postoperative day 1. (**A**) The arrow indicates contrast accumulation observed on the left side of the neck. (**B**) A CT scan performed immediately afterward does not show any contrast accumulation and increased focal fatty tissue density is observed. The arrow in (**B**) points to the same location as in (**A**).

**Figure 3:**
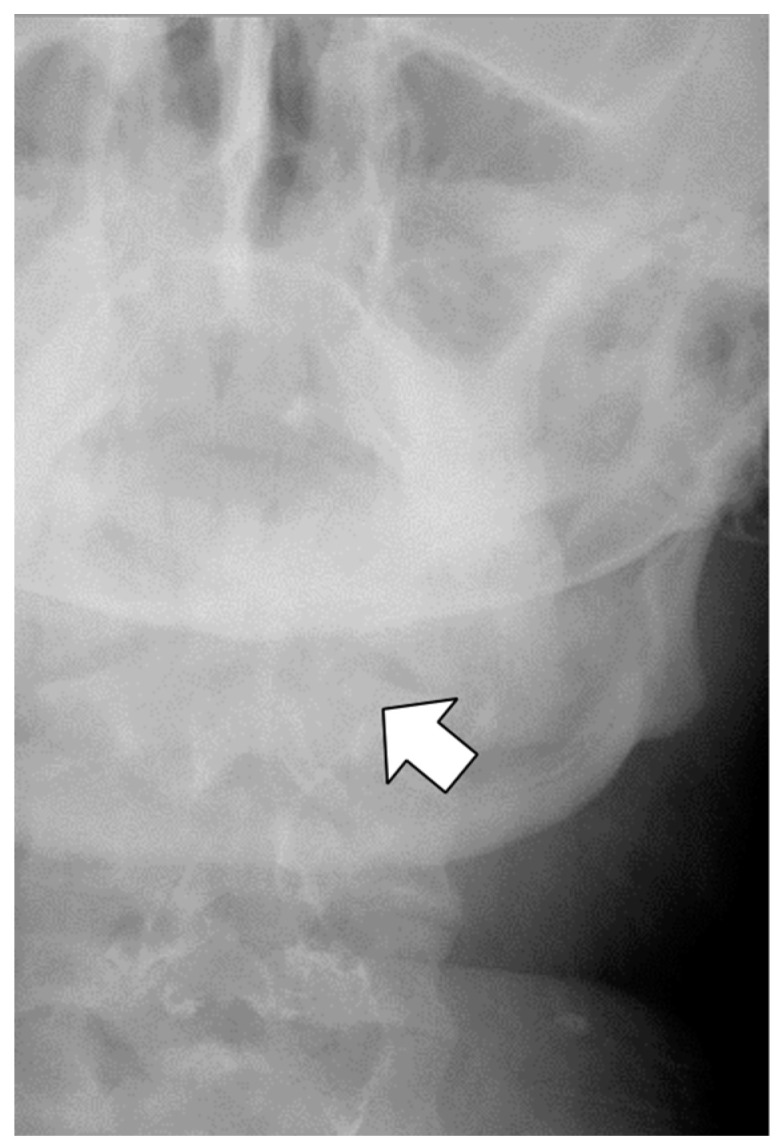
The arrow indicates no contrast retention is observed at the left pisiform fossa on postoperative day 4.

Antibiotics (sulbactam–ampicillin, 3 g every 8 h, up to postoperative day 3) were administered to prevent infection of oral anaerobic bacteria. On postoperative day 1, the patient complained of left neck pain, which was managed with acetaminophen as needed. Further, blood exams showed a white blood cell count (WBC) of 21,300/μL and a C-reactive protein (CRP) level of 5.76 mg/dL, and the patient was afebrile. Fluoroscopy on postoperative day 1 revealed contrast retention in the left lateral neck, indicating submucosal leakage (Figure 2). Thus, a CT scan was performed immediately, which did not show any contrast accumulation in the left lateral neck; therefore, we believed that there was no prolonged retention of the leaked fluid. In addition, we observed a focal escalation of fatty tissue density on the left side of the neck and confirmed that there was no spread of inflammation to the mediastinum. Based on these results, we considered that the inflammatory spillover to the surrounding area due to saliva retention was minor and determined that conservative treatment could be continued. While continuing oral clear water intake, WBC count and CRP levels improved on postoperative day 3 to 13,610/μL and 2.23 mg/dL, respectively. Fever was not observed during the study period. The pain in the left neck gradually subsided, and no pain was noted on postoperative day 3. Fluoroscopy performed on postoperative day 4 confirmed no submucosal leakage of the contrast medium (Figure 3). The nasogastric tube was removed on postoperative day 4. Finally, the patient was discharged from the hospital on postoperative day 6. The patient visited the hospital 3 days after discharge (postoperative day 9) and was confirmed to have no fever or elevated inflammatory response on blood examination. Thereafter, follow-ups were conducted according to our weight-loss program. Her weight decreased to 74.8 kg (BMI, 32.5 kg/m^2^, percent total weight loss, 23.1%) 17 months after the surgery, without complaints of neck pain or discomfort during swallowing.

## 3. Discussion

The piriform fossa is a pharyngeal depression. Injury to this area can cause significant discomfort, and potentially lead to complications such as bleeding, infection, or even full-layer perforation.

An iatrogenic piriform fossa or esophageal injury during LSG is an uncommon complication [5]. Our literature search did not identify any studies that specifically reported the incidence rate of piriform fossa and/or esophageal injuries caused by calibration tube insertion in BMSs. Compared to the frequency of iatrogenic perforation in gastrointestinal endoscopy, which is estimated to be 0.03–0.8%, the actual situation may be underestimated [10].

Typically, during LSG, a dedicated calibration tube or endoscope is inserted orally into the stomach through the pharynx and esophagus. In Japan, there is no pharmaceutically approved calibration tube specifically designed for LSG, and intraoperative endoscopy is not always feasible. Consequently, inexpensive natural rubber catheters are often used instead of dedicated calibration tubes. In the present case, a 36-Fr Nelaton catheter was used as the calibration tube, as per usual practice. Unfortunately, the injury occurred during the procedure, which was our hospital’s first incident out of 204 procedures performed.

Although piriform fossa and/or esophageal injuries during LSG can lead to severe adverse outcomes, only a few cases regarding them have been reported [11]. We searched PubMed for studies that described perforation of the gastrointestinal tract during LSG using the keywords “bariatric surgery”, “perforation”, and “bougie”, and found six cases reported in five articles (Table 1) [9,12,13,14,15]. In all reported cases, surgical intervention, such as primary closure, patch and reinforcement with the surrounding connective tissue, and placement of a drainage tube near the injured area, was required. The definitive difference between previous cases and ours is the depth of the injury. In previous reports, all cases had full-layer injuries; however, in our case, the depth of the perforation was limited to the submucosa, which is the first such case described in the literature. Hence, it was assumed that it could be treated conservatively, similar to not requiring suture closure in endoscopic submucosal dissection. Surgical interventions, such as suture reinforcement or patching, should be preferred if the perforation is deeper than the mucosal layer.

Fortunately, in this case, we were able to detect the injury intraoperatively, facilitating prompt intervention. According to other literature, the survival rate of esophageal injuries improves when interventions are performed within 12–24 h of occurrence. However, this report was not recently published, and medical care standards have likely evolved since [16,17]. In cases of postoperative hypoxia, neck pain, dysphagia, and dyspnea, it is important to consider the possibility of piriform fossa and/or esophageal injury and evaluate the need for imaging studies such as fluoroscopy or CT as mentioned in previous reports [9,12,14]. Fever and tachycardia can also indicate abnormal postoperative progression.

The causes of these injuries can be diverse, including anatomical abnormalities, mainly the three physiological esophageal narrowing sites (the cricopharyngeal sphincter, aortic arch, and lower esophageal sphincter), as well as factors attributable to obesity, poor calibration tube insertion technique, and the shape or rigidity of the calibration tubes. An upper gastrointestinal endoscopy was performed preoperatively to evaluate the presence of GERD, hiatal hernia, and esophageal/gastric cancer. During the preoperative endoscopy, no anatomical abnormalities were observed in the pharynx, larynx, or upper esophagus, and the endoscope was easily inserted. In previous reports, it was unclear whether preoperative evaluations, such as upper gastrointestinal endoscopy or CT, were performed [9,12,13,14,15]. While we believe that these assessments are necessary to reduce risks, the absence of examination abnormalities does not guarantee complete avoidance of injuries, as demonstrated in this case.

The anesthesiologist in charge of this case had 4 years of experience in anesthesiology and a total of approximately 20 cases of experience in administrating anesthesia in BMS. The patient had Mallampati classification class 3 and Cormack classification grade 1 and was at a relatively high risk of difficult intubation. The patient underwent esophageal intubation, which required reintubation using a video-blade laryngoscope. Patients with severe obesity are often difficult to intubate because of their short necks, small jaws, and impaired mouth openings, which may contribute to iatrogenic injuries. Furthermore, it should be noted that when the patient is intubated, the intubation tube pressurizes the pharyngeal cavity and the piriform fossa, increasing the risk of injury.

Therefore, perforation was more likely to be caused by the technique of calibration tube insertion rather than by pre-existing anatomical abnormalities. Our recommendation is to use a video-blade laryngoscope for patients in whom insertion of a calibrated tube is expected to be extremely difficult. Blind calibration tube insertion should be performed with caution as there is a potential risk of injury or perforation. Therefore, it is crucial to remain vigilant regarding these risks. In addition, we strongly advise checking the situation and attempting to reinsert the tube with the assistance of a video blade laryngoscope upon encountering excessive resistance, as in this case, to prevent or minimize any further potential damage.

In addition to the aforementioned technical concerns, the shape and rigidity of the calibration tube should be considered, and the use of a catheter specifically designed for LSG is desirable.

Currently, there are no standardized guidelines for treating iatrogenic esophageal injuries caused by calibrated tube insertion. In our case, intraoperative endoscopy using carbon dioxide showed that the cavity was easily collapsed by endoscopic insufflation; therefore, we judged that the possibility of physical retention of saliva was low and adopted a conservative treatment regimen. Moreover, we performed nasogastric tube insertion, which we believed would reduce saliva flow into the perforated dead space, functioning as a guiding tube, such as a splint. Fortunately, our patient could be treated conservatively and did not develop subsequent neck abscesses or mediastinitis. However, surgical drainage, patching, and other surgical interventions are often required [9,12,13,14,15]. Patients with clinically severe obesity, who often have short, thick necks and coexisting diabetes mellitus, may have higher risks for severe neck abscesses and airway obstruction, as well as mediastinitis and mediastinal abscesses. Therefore, despite opting for conservative treatment, it is critical to monitor patient progression closely during the postoperative period.

The limitation of this study is that it is based on our single experience. We have not been able to provide evidence to assert that the submucosal injury with calibration tube insertion can be treated conservatively in all cases, as in our case. Previous reports did not mention treatment for injuries that remained in the submucosal layer; however, we believe that this report raises the possibility of considering conservative treatment depending on the severity of the injury.

The frequency of pisiform fossa and/or esophagus injuries remains unknown, highlighting the need for comprehensive multicenter surveys on these injuries in the future. We hope that such studies can provide invaluable insights into the incidence and prevalence of these injuries, as well as their underlying risk factors and potential prevention and treatment strategies.

## 4. Conclusions

We report the case of an intraoperative iatrogenic injury to the piriform fossa during LSG, which occurs rarely; however, given that it may lead to serious complications, the risk should not be underestimated. Although calibration tube insertion appears simple, special care should be taken because of the blinded nature of the technique; surgeons must be aware of this possibility and take necessary precautions to avoid such complications.

Conservative management is possible in selected cases; however, prompt recognition and intervention are essential to prevent serious consequences.

## Figures and Tables

**Table 1:** Previous reports and our case of injuries related to calibration tube insertion.

Author	No. of Cases Reported	Age	Sex	Preoperative BMI	Procedure	Presentation	Diagnosed	Injury Site	Treatment	Hospitalized Days after Intervention	Outcome	Latest Observation	Abnormality of the Perforated Area
Tadayon SMK [12]	1	33	F	41.5	LSG	Respiratory distress and subcutaneous emphysema on the right side of the head and neck	1POD	The left side of the posterior wall of the cervical esophagus	Primary closure	10	Alive	3M	None
									Enterostomy				
Patel A [13]	1	66	F	46.7	LRYGB	-	intra-operative	The posterior perforation at the distal esophagus	Primary closure and drainage	4	Alive	N/A	N/A
Lovece A [14]	1	42	F	31	LSG	Chest discomfort	1POD	The posterior wall of the cervical esophagus	Primary closure and drainage	8	Alive	N/A	N/A
Signorini FJ [15]	1	64	F	39	LSG	-	intra-operative	Below the arch of the azygos vein	Primary closure	6	Alive	2Y	None
Theodorou D [9]	2	56	F	44.5	LSG	Mild discomfort and dyspnea	5POD	The lower esophagus	Total gastrectomy, cervical esophagostomy, and enterostomy	28	Alive	N/A	N/A
		41	F	48	LAGB	Left pleuritic pain	2POD	middle esophagus	Total esophagectomy, cervical esophagostomy and enterostomy	18	Dead	N/A	N/A
Our case	1	36	F	42.3	LSG	-	Intraoperative	The left piriform fossa	Conservative (nasogastric tube, antibiotics)	6	Alive	17M	None

BMI, Body mass index; LSG, Laparoscopic Sleeve Gastrectomy; LRYGB, Laparoscopic Roux-En-Y Gastric Bypass; LAGB, Laparoscopic Adjustable Gastric Banding; POD, postoperative day, N/A; not available.

## Data Availability

No new data were created or analyzed in this study. Data sharing is not applicable to this article.
